# The genetic architecture of the association between eating behaviors and obesity: combining genetic twin modeling and polygenic risk scores

**DOI:** 10.1093/ajcn/nqaa181

**Published:** 2020-07-20

**Authors:** Guiomar Masip, Karri Silventoinen, Anna Keski-Rahkonen, Teemu Palviainen, Pyry N Sipilä, Jaakko Kaprio, Leonie H Bogl

**Affiliations:** Department of Public Health, University of Helsinki, Helsinki, Finland; Department of Public Health, University of Helsinki, Helsinki, Finland; Population Research Unit, Faculty of Social Sciences, University of Helsinki, Helsinki, Finland; Department of Public Health, University of Helsinki, Helsinki, Finland; Institute for Molecular Medicine Finland (FIMM), University of Helsinki, Helsinki, Finland; Department of Public Health, University of Helsinki, Helsinki, Finland; Department of Public Health, University of Helsinki, Helsinki, Finland; Institute for Molecular Medicine Finland (FIMM), University of Helsinki, Helsinki, Finland; Institute for Molecular Medicine Finland (FIMM), University of Helsinki, Helsinki, Finland; Department of Epidemiology, Center for Public Health, Medical University of Vienna, Vienna, Austria

**Keywords:** eating behavior patterns, diet quality score, polygenic risk score, obesity measures, susceptibility to obesity, heritability, twins, genetics, BMI, snacking

## Abstract

**Background:**

Obesity susceptibility genes are highly expressed in the brain suggesting that they might exert their influence on body weight through eating-related behaviors.

**Objectives:**

To examine whether the genetic susceptibility to obesity is mediated by eating behavior patterns.

**Methods:**

Participants were 3977 twins (33% monozygotic, 56% females), aged 31–37 y, from wave 5 of the FinnTwin16 study. They self-reported their height and weight, eating behaviors (15 items), diet quality, and self-measured their waist circumference (WC). For 1055 twins with genome-wide data, we constructed a polygenic risk score for BMI (PRS_BMI_) using almost 1 million single nucleotide polymorphisms. We used principal component analyses to identify eating behavior patterns, twin modeling to decompose correlations into genetic and environmental components, and structural equation modeling to test mediation models between the PRS_BMI_, eating behavior patterns, and obesity measures.

**Results:**

We identified 4 moderately heritable (*h*^2^ = 36–48%) eating behavior patterns labeled “snacking,” “infrequent and unhealthy eating,” “avoidant eating,” and “emotional and external eating.” The highest phenotypic correlation with obesity measures was found for the snacking behavior pattern (*r* = 0.35 for BMI and *r* = 0.32 for WC; *P* < 0.001 for both), largely due to genetic factors in common (bivariate *h*^2^ > 70%). The snacking behavior pattern partially mediated the association between the PRS_BMI_ and obesity measures (*β*_indirect_ = 0.06; 95% CI: 0.02, 0.09; *P* = 0.002 for BMI; and *β*_indirect_ = 0.05; 95% CI: 0.02, 0.08; *P* = 0.003 for WC).

**Conclusions:**

Eating behavior patterns share a common genetic liability with obesity measures and are moderately heritable. Genetic susceptibility to obesity can be partly mediated by an eating pattern characterized by frequent snacking. Obesity prevention efforts might therefore benefit from focusing on eating behavior change, particularly in genetically susceptible individuals.

See corresponding editorial on page 913.

## Introduction

Obesity is influenced by a wide range of genetic and environmental factors (1, 2). Substantial evidence suggests that the overconsumption of energy-dense foods and drinks is an important etiological factor for weight gain and obesity (3). In addition, eating behaviors and diet quality have been associated with overweight and weight gain in several studies (4–9), hence they might represent potential key pathways that could help to explain the genetic susceptibility to obesity. Twin studies have not only shown that ∼57–90% of the variance in adult BMI (2, 10–12) and 40–60% of the variance in eating behavioral traits (including dietary intake) is attributable to genetic factors (13–15), but have also suggested that the associations between eating behavior traits, such as cognitive restraint, uncontrolled eating, and emotional eating, and BMI are partly mediated by genetic factors common to both (16).

Recent advances in genetic studies have increased our knowledge of common susceptibility genetic loci associated with obesity. The meta-analysis of genome-wide association studies (GWASs) of BMI from Locke et al. (17) identified 97 BMI-associated loci [single nucleotide polymorphisms (SNPs)], but explained <4% of the BMI variance. A more recent meta-analysis of GWASs by Yengo et al. (18) extended the number of BMI-associated SNPs to 751, explaining 6% of the variance in BMI. Whole-sequence genome data increase the fraction of variance of BMI accounted for by genetic variants, both common and rare, to 40% (). The pathways through which obesity-associated genetic variants predispose certain individuals to develop obesity are not well understood, but because obesity susceptibility genes are highly expressed in the central nervous system (19), they are likely to influence appetitive and satiety traits, which therefore represent a plausible behavioral pathway for weight gain (16, 20, 21).

A few earlier studies have focused on the associations between eating behaviors and genetic susceptibility to obesity. Two studies performed in adults have reported that uncontrolled eating and emotional eating mediate the association between a polygenic risk score (PRS) comprising 90 BMI-associated loci and obesity measures (21, 22). A more recent study that used a 97-loci PRS indicated that the genetic susceptibility to obesity is partially mediated by disinhibition and susceptibility to hunger (23). These previous studies have defined genetic susceptibility to obesity based on <100 SNPs that reached genome-wide significance. Khera et al. (24) recently suggested the use of a PRS by incorporating all available information from ≤2.1 million common genetic variants irrespective of genome-wide significance, hence providing a more powerful approach for capturing genetic risk (18). Research has not yet determined the associations between such a whole-genome–based PRS for obesity, eating behavior patterns, and obesity measures. Thus, the aims of this study were to examine whether eating behavior patterns are related to obesity measures, to study whether genetic susceptibility to obesity is mediated by eating behavior patterns in a nationwide setting in Finland using the latest PRS for obesity, and to test these associations using a genetically informative twin design.

## Methods

### Participants

**FIGURE 1:**
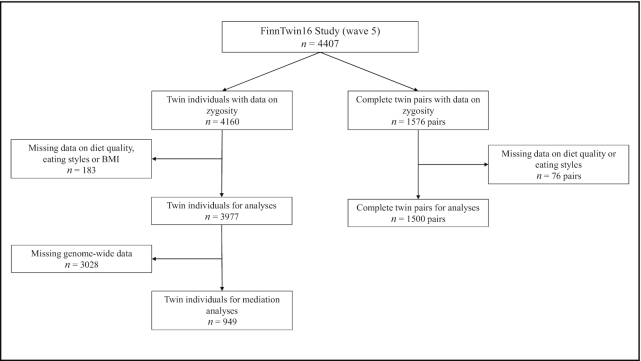
Flowchart diagram of participants’ selection.

The data for this study were derived from the longitudinal FinnTwin16 (FT16) cohort including Finnish twins born in 1975–1979 and identified from the population register (*n* = ∼5600 twin individuals) (25). The FT16 study was targeted to investigate determinants of health-related behaviors, disease risk factors, and chronic diseases in adolescents and young adults. Data from the latest survey in 2010–2012 (wave 5), when the participants were on average 34 y old, were used in the present study. The questionnaire was administered as an internet survey. The invitation was sent to all twins belonging to the original target population, regardless of earlier participation (total *n* = 4407 twin individuals, response rate 72%, and *n* = 1055 twin individuals had genome-wide data available). We excluded participants with unknown zygosity, eating styles, diet quality, and BMI. In mediation analyses we excluded twin individuals with missing genome-wide data. In quantitative twin genetic analyses, we excluded those without a co-twin and without information on zygosity, eating styles, and diet quality. The final data (**Figure 1**) included 3977 twin individuals [527 monozygotic (MZ), 470 same-sex dizygotic (SS-DZ), and 503 opposite sex-dizygotic (OS-DZ) twin pairs] (**Supplemental Table 1**A) and 949 twin individuals for mediation analyses (**Supplemental Table 1**B). Zygosity was derived from questionnaires, based on physical similarity and confusing by others, a method that has shown high reliability in this cohort (26). The data collection was approved by the Ethics Committee of the Central Finland Health Care District (April 20, 2010, Dnro 4/2010).

### Measurements

#### Eating styles and diet quality

Eating styles were assessed with a 16-item questionnaire modified from Keski-Rahkonen et al.(27). The questionnaire was cross-validated with various established assessment tools—the Three-Factor Eating Questionnaire (28, 29), the Binge Eating Scale (30), and the Eating Disorder Inventory (31)—in a small subsample (*n* = 42) of FinnTwin16 participants. Subscales of those instruments were significantly correlated with our eating style items except for those describing health-conscious/avoidant eating and 1 item regarding snacking. However, in the study from Keski-Rahkonen et al. (27), health-conscious eating and snacking items had moderate internal consistency (Cronbach α = 0.70 and 0.61, respectively). The participants were asked to select the option that best described their overall eating style (2 items for assessing meal frequency and 2 items for regular eating styles). Another 3 items inquired about health-conscious eating, 1 item about night eating, 1 item about external eating, 2 items about emotional eating, and 5 items about snacking styles with 4 different alternatives: “usually,” “often,” “sometimes,” or “seldom” (32). The item about night eating style was excluded due to a lack of variability in participants’ responses. Dietary intake was assessed by a short FFQ, from which a diet quality score (DQS) was calculated, as previously described (32). The DQS had been previously validated and is associated with obesity measures and eating styles in this cohort (32). The DQS ranges from 0 to 12 points, with a higher score indicating a better diet quality.

#### Obesity measures

Height and weight were self-reported, from which BMI (kg/m^2^) was calculated. Waist circumference (WC) was self-measured midway between the lowest rib and the iliac crest with a tape. The participants received a tape and instructions by mail on how to self-measure WC. The validity of self-reported height, weight, and WC was previously demonstrated in a subsample of FT16 participants (33).

#### Genotyping and weighted PRS

Genotyping was performed in the United States, United Kingdom, and Finland (see **Supplemental Methods** for more details). Genotyping quality control batches were applied for SNPs [minor allele frequency (MAF)]. First, we removed every genetic variant with a call rate <0.975 (batches 1 and 3) or <0.95 (batch 2). Second, we removed every sample with a call rate <0.98 (batch 1) or <0.95 (batches 2 and 3). Finally, variants were filtered by their MAF <0.01 with Hardy–Weinberg equilibrium *P* value <1e-06. Samples from all batches with heterozygosity test method-of-moments *F* coefficient estimate values <-0.03 or >0.05 (batches 1 and 2) or ±4 SD from the mean (batch 3) were removed, along with the samples that failed the sex check or were among the multidimensional scaling principal component analysis outliers. Genotyped autosomal variants after quality control were 475,526 (batch 1), 239,894 (batch 2), and 388,673 (batch 3), with the following number of samples remaining for imputation: 2617 (batch 1), 5328 (batch 2), and 8218 (batch 3).

Prephasing of the data was done with Eagle v2.3 (34) and imputation with Minimac3 v2.0.1 using the University of Michigan Imputation Server (35). Genotypes from all the batches were imputed to the Haplotype Reference Consortium release 1.1 reference panel (36).

#### Polygenic scoring

Genetic susceptibility was assessed by calculating PRSs using the Bayesian approach accounting for linkage disequilibrium (LD) between each genetic variant (37). We did not use any pruning and thresholding method to select genetic variants. The infinitesimal model for polygenic scoring was adjusted by an LD reference panel consisting of the Finnish FINRISK study (*n* = 27,284) (38). GWAS summary statistics and the FT16 study sample were restricted to the European HapMap3 variants (39) with an MAF >5%. The major histocompatibility complex gene cluster of human chromosome 6 (GRCh37: 6p22.1–21.3) was excluded due to strong LD block. We derived 2 PRSs, 1 for BMI (PRS_BMI_) and 1 for waist-to-hip ratio (WHR) adjusted for BMI (PRS_WHR_). The total numbers of genetic variants used for the PRS calculations were 996,919 for BMI and 1,148,565 for WHR adjusted for BMI, with the reweighted effect sizes available from 692,578 and 484,563 samples, respectively (see Supplemental Methods for more details of the different PRSs). The PRS_BMI_ explained 8.3% of the variance in BMI in this study sample.

### Statistical methods

General characteristics of the study sample are presented as means and 95% CIs for continuous variables and as numbers and percentages for categorical variables. Differences between men and women were determined using the adjusted Wald test for continuous variables and Pearson χ^2^ test for categorical variables.

#### Principal component analyses

Eating behavior patterns were derived by principal component analyses (PCAs) using 15 eating styles and 1 indicator of diet quality (DQS). Based on eigenvalues >1.0 and scree plot analyses, we retained 4 components. We calculated factor loadings after a varimax rotation to simplify and facilitate their interpretability. Factor loadings (≥0.30) were considered to contribute to the eating behavior pattern and were used to label the 4 components.

#### Quantitative genetic analyses

We used quantitative genetic twin modeling, based on linear structural equations, to estimate the heritability of eating behavior patterns and obesity measures (40). Classical twin modeling is based on the assumption that MZ twins share virtually the same gene sequence, whereas dizygotic (DZ) twins share, on average, 50% of their genetic variance, like other siblings. Based on these assumptions, genetic variation can be decomposed into additive genetic variation (A), which is the sum of the allelic effects on the phenotype over all relevant loci, and dominance genetic variation (D), including nonadditive genetic effects. Environmental variation can be decomposed into shared environmental variation (C), which includes all environmental factors that make co-twins similar, and nonshared environmental variation (E), which includes all environmental factors that make co-twins dissimilar (as well as measurement error). The expected correlations for additive and dominance genetic effects are both 1 for MZ twins and 0.5 and 0.25 for DZ twins, respectively. By definition, the expected correlations for shared and nonshared environmental effects are 1 and 0, respectively, for both MZ and DZ twins. Before model fitting, we calculated intraclass correlations (ICCs) for MZ, SS-DZ, and OS-DZ twin pairs to estimate the level of within-pair similarity and to provide evidence for the presence of genetic effects (40).

We started the analyses by computing univariate models (41). Because we had information only on twin pairs reared together, we were unable to estimate dominance genetic and shared environmental effects simultaneously. Thus, we first tested whether shared environmental factors and dominant genetic factors were present to explain the variation in eating behavior patterns and obesity measures by comparing the ACE and ADE models with the AE model. No evidence of C or D was found; hence, a more parsimonious additive genetic/nonshared environment (AE) model was used in all further genetic twin modeling.

After the univariate models, we calculated correlations between eating behavior patterns and obesity using Pearson partial correlations. Then we used the bivariate Cholesky decomposition to examine the extent to which genetic and nonshared environmental effects underlie the observed correlation between the 2 traits. In addition, this bivariate design allowed us to derive additive genetic (*r*_a_) and shared environmental (*r*_c_) correlations (40). All analyses were adjusted for age and sex.

#### Structural equation modeling

Structural equation modeling (SEM) analyses were conducted to identify potential mediation models between the PRSs (PRS_BMI_ and PRS_WHR_) and obesity measures through eating behavior patterns (42). To control for age, sex, and population stratification, we regressed all of the variables on age, sex, and genetic principal components and then used the residuals in the SEM analyses. Genetic principal components came from the merged raw phenotype data of all samples, to detect any population stratification due to systematic ancestry differences (43). Analyses with PRS_WHR_ were only controlled for age and sex. Total effects (c + ab), direct effects (c), and indirect or mediation effects (ab) were calculated to show how much of the association between the PRS and the obesity measure was mediated by the different eating behavior patterns. Bias corrected estimates and 95% CIs were calculated using the bootstrapping approach with 1000 draws. All of the models were estimated for every eating behavior pattern and for both obesity measures using both PRSs, and the results are presented as total, direct, and indirect effects. We added interaction terms to test whether the mediation models were similar in men and women. Because some of the interaction terms were significant (*P* < 0.05), we present the mediation models separately by sex in **Supplemental Table 3**.

When analyzing twins as individuals, the effect of the twin pair clustering was taken into account in SEs yielded by cluster variance estimators, which were robust to nonindependent observations within families (44). The descriptive statistics, PCAs, ICCs, Pearson partial correlations, and SEM were conducted by using Stata statistical software (release 14.1; StataCorp). Quantitative genetic analyses were carried out with the OpenMx package (version 2.14.11) of R statistical software (R Project for Statistical Computing) (45). A *P* value <0.05 was considered as the level of significance.

## Results

### General characteristics of the study sample

**Table 1** shows the descriptive characteristics of the study sample. Approximately 56% of the study sample were women and 33% were MZ twins. The participants had, on average, a BMI of 24.8 and a WC of 86.1 cm. The mean DQS was 7 points from a maximum of 12 points. Women had a lower BMI and WC, but they were abdominally obese more often than men. Women had a higher diet quality than men (mean DQS: 7.4 for women and 6.4 for men; *P* < 0.001). There were no differences between men and women with respect to standardized PRSs. Supplemental Table 1A shows the descriptive statistics by zygosity, and Supplemental Table 1B shows the descriptive statistics for the subsample used in the mediation analyses.

**FIGURE 3:**
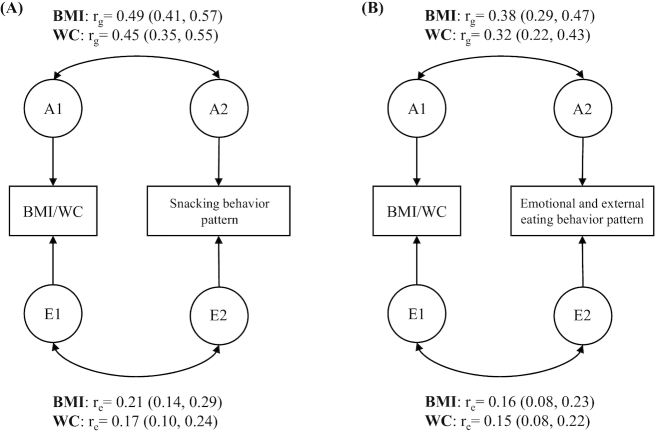
(A) Path diagram of a bivariate Cholesky model for 1 member of a twin pair. Additive genetic (*r*_a_) and nonshared environmental (*r*_e_) correlations between BMI/WC and the snacking behavior pattern and 95% CIs. The covariance of the traits was decomposed into additive genetic (A) and nonshared environmental (E) effects. All models were adjusted for age and sex; *n* = 527 monozygotic, 470 same-sex dizygotic, and 503 opposite-sex dizygotic pairs. (B) Path diagram of a bivariate Cholesky model for 1 member of a twin pair. Additive genetic (*r*_a_) and nonshared environmental (*r*_e_) correlations between BMI/WC and the emotional and external eating behavior pattern and 95% CIs. The covariance of the traits was decomposed into additive genetic (A) and nonshared environmental (E) effects. All models were adjusted for age and sex; *n* = 527 monozygotic, 470 same-sex dizygotic, and 503 opposite-sex dizygotic pairs. WC, waist circumference.

**TABLE 1:** General characteristics of the study sample^1^

Characteristics	Overall (*n* = 3977)	Men (*n* = 1732)	Women (*n* = 2245)	*P* value
Monozygotic twins, *n* (%)	1298 (32.6)	505 (29.2)	793 (35.3)	0.001
Same-sex dizygotic twins, *n* (%)	1272 (32.0)	603 (34.8)	669 (29.8)	
Opposite-sex dizygotic twins, *n* (%)	1407 (35.4)	624 (36.0)	783 (34.9)	
Age, y, mean (95% CI)	34.1 (34.1, 34.1)	34.1 (34.1, 34.2)	34.1 (34.0, 34.1)	0.4
BMI, kg/m^2^, mean (95% CI)	24.8 (24.6, 24.9)	25.7 (25.5, 25.9)	24.0 (23.8, 24.2)	<0.001
Obesity (BMI >30 kg/m^2^), *n* (%)	415 (10.4)	186 (10.7)	229 (10.2)	0.6
WC,^2^ cm, mean (95% CI)	86.1 (85.6, 86.5)	92.1 (91.6, 92.7)	81.2 (80.7, 81.8)	<0.001
Abdominal obesity (women >88 cm; men >102 cm),^2^*n* (%)	771 (20.1)	268 (15.8)	503 (23.6)	<0.001
Diet quality score, mean (95% CI)	7.0 (6.9, 7.0)	6.4 (6.3, 6.5)	7.4 (7.3, 7.5)	<0.001
Standardized PRS_BMI_,^2^ mean (95% CI)	6.6e−10 (−0.1, 0.1)	0.0 (−0.1, 0.2)	0.0 (−0.1, 0.1)	0.4
Standardized PRS_WHR_,^2^ mean (95% CI)	1.2e−09 (−0.1, 0.1)	0.0 (−0.1, 0.2)	0.0 (−0.1, 0.1)	0.4

1 Differences between men and women, *P* values, were determined by the adjusted Wald test for continuous variables and Pearson χ^2^ test for categorical variables and corrected for the clustering of twin pairs by survey methods. PRS, polygenic risk score; WC, waist circumference; WHR, waist-to-hip ratio.

2 Sample size smaller due to missing genotypes. Overall sample size for WC and abdominal obesity *n* = 3832; overall sample size for standardized PRS_BMI_ and standardized PRS_WHR_*n* = 949.

### Eating behavior patterns

Results of the PCAs of eating behavior patterns, which included 15 questions on eating styles and the DQS, are shown in **Table 2**. The resultant eating behavior patterns were labeled “snacking,” “infrequent and unhealthy eating,” “avoidant eating,” and “emotional and external eating.” These 4 components together explained ∼56% of the variance in eating styles and diet quality.

**TABLE 2:** Eating behavior patterns and factor loadings in varimax-rotated principal components

		Snacking	Infrequent and unhealthy eating	Avoidant eating	Emotional and external eating
Eigenvalue		4.00	2.43	1.38	1.09
Percentage variance explained		16.6	13.6	12.8	12.7
Variables	Response coding				
Diet quality score	From healthy to unhealthy	0.00	0.33	0.24	0.04
How often do you eat breakfast?	From usually to seldom	0.07	0.50	−0.03	−0.04
How often in a day do you usually eat?	From usually to seldom	-0.18	0.58	−0.06	0.01
Regularity of your eating habits	From regular to irregular	0.28	0.36	−0.02	−0.09
Alternating restriction and overeating style	From seldom to usually	0.33	0.02	−0.20	0.07
During meals I eat sufficiently—I don't need to snack	From usually to seldom	0.52	−0.19	0.05	−0.07
I replace my meals with snacks	From seldom to usually	0.45	0.05	−0.07	−0.11
I eat most in the evenings	From seldom to usually	0.28	0.19	−0.01	0.14
I usually munch constantly in the evenings	From seldom to usually	0.38	−0.06	0.06	0.14
I tend to eat healthily	From usually to seldom	0.19	0.14	0.36	−0.05
I avoid greasy meals	From usually to seldom	0.02	0.01	0.60	0.04
I avoid calories	From usually to seldom	−0.04	−0.08	0.63	−0.02
While I am eating, I watch TV	From seldom to usually	−0.02	0.22	−0.05	0.40
I am tempted to eat according to advertisements	From seldom to usually	−0.08	0.04	0.04	0.55
I reward myself with good food	From seldom to usually	−0.02	−0.08	−0.01	0.56
I console myself by eating or drinking	From seldom to usually	0.17	0.13	−0.01	0.40

Sample size *n* = 3977.

### ICCs and heritability estimates

**FIGURE 2:**
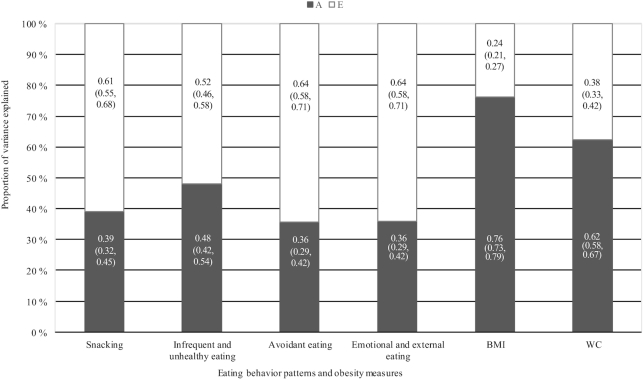
Proportion of variation of the eating behavior patterns and obesity measures explained by additive genes (A) and nonshared environmental factors (E). The numbers within the bars are means and 95% CIs; *n* = 3000. WC, waist circumference.

ICCs were higher in MZ twins than DZ twins, indicating the presence of genetic influences (Supplemental Table 2). Heritability estimates of eating behavior patterns and obesity measures are shown in **Figure 2**. The variation in eating behavior patterns and obesity measures was due to both additive genetic and nonshared environmental factors. Eating behavior patterns showed moderate heritability estimates, ranging from 36% to 48%. As expected, the heritability estimates for BMI and WC were high (76% and 62%, respectively). When stratifying the analyses by sex, heritability estimates were slightly higher in women than in men, although CIs were overlapping (**Supplemental Figure 1**).

### Associations between eating behavior patterns and obesity measures

All of the 4 eating behavior patterns were positively correlated with obesity measures, except for the avoidant eating behavior pattern, which was not correlated with BMI, and the infrequent and unhealthy eating behavior pattern, which was not correlated with WC adjusted for BMI (**Table 3**). For both BMI and WC, the highest phenotypic correlations were observed for the snacking behavior pattern (*r*_p_ = 0.35 and 0.32, respectively) and the emotional and external eating behavior pattern (*r*_p_ = 0.26 and 0.23, respectively). Thus, we decomposed these 4 correlations into genetic and environmental components (**Figure 3**A, B). Higher scores on the snacking and avoidant eating behavior patterns were associated with a higher WC adjusted for BMI, but these associations were very weak (Table 3). Therefore, we did not decompose these correlations into genetic and environmental components.

**TABLE 3:** Association between eating behavior patterns and obesity measures

Obesity measures		Pearson partial correlation	
	Eating behavior patterns	*r*	*P* value
Body mass index	Snacking	0.35	<0.001
	Infrequent and unhealthy eating	0.12	<0.001
	Avoidant eating	0.01	0.8
	Emotional and external eating	0.26	<0.001
Waist circumference	Snacking	0.32	<0.001
	Infrequent and unhealthy eating	0.11	<0.001
	Avoidant eating	0.04	0.02
	Emotional and external eating	0.23	<0.001
Waist circumference adjusted for BMI	Snacking	0.06	0.001
	Infrequent and unhealthy eating	0.02	0.2
	Avoidant eating	0.07	<0.001
	Emotional and external eating	0.03	0.09

Figure 3A shows the path diagram of the bivariate Cholesky model decomposing the correlations between the snacking behavior pattern and obesity measures (BMI and WC), and Figure 3B shows the corresponding results for the emotional and external eating behavior pattern and obesity measures (BMI and WC). For the snacking and emotional and external eating behavior patterns, the additive genetic correlations with both obesity measures were higher than the nonshared environmental correlations. The proportion of the covariance explained by genes was 75% (95% CI: 61, 88%) and 71% (95% CI: 58, 93%) for the snacking behavior pattern and BMI and WC, respectively; and 75% (95% CI: 55, 87%) and 64% (95% CI: 44, 85%) for the emotional and external eating behavior pattern and BMI and WC, respectively.

### Eating behavior patterns as mediators of genetic susceptibility to obesity

As expected, the PRS_BMI_ was positively associated with BMI and WC. Among the 4 eating behavior patterns, only the snacking and the infrequent and unhealthy eating behavior patterns significantly mediated the association between the PRS_BMI_ and both obesity measures (BMI and WC) (**Table 4**). However, the infrequent and unhealthy eating behavior pattern mediated this association to a much weaker extent (see Supplemental Figure 2 for more details). The emotional and external eating behavior pattern only mediated the association between the PRS_BMI_ and BMI.

**TABLE 4:** Structural equation modeling of the PRS_BMI_ and obesity measures^1^

	BMI (*n* = 949)		WC (*n* = 874)	
	*β* (95% CI)	*P* value	*β* (95% CI)	*P* value
Mediation model through snacking				
Total effect of PRS_BMI_	0.29 (0.20, 0.38)	<0.001	0.24 (0.15, 0.32)	<0.001
Direct effect of PRS_BMI_	0.23 (0.16, 0.31)	<0.001	0.19 (0.12, 0.26)	<0.001
Indirect effect (via snacking) of PRS_BMI_ on obesity measures	0.06 (0.02, 0.09)	0.002	0.05 (0.02, 0.08)	0.003
Percentage mediation	20.7		20.8	
Mediation model through infrequent and unhealthy eating				
Total effect of PRS_BMI_	0.29 (0.20, 0.38)	<0.001	0.24 (0.15, 0.32)	<0.001
Direct effect of PRS_BMI_	0.28 (0.20, 0.36)	<0.001	0.23 (0.15, 0.31)	<0.001
Indirect effect (via infrequent and unhealthy eating) of PRS_BMI_ on obesity measures	0.01 (0.00, 0.02)	0.04	0.01 (0.00, 0.02)	0.04
Percentage mediation	3.4		4.2	
Mediation model through avoidant eating				
Total effect of PRS_BMI_	0.29 (0.20, 0.38)	<0.001	0.24 (0.15, 0.32)	<0.001
Direct effect of PRS_BMI_	0.29 (0.21, 0.38)	<0.001	0.24 (0.16, 0.32)	<0.001
Indirect effect (via avoidant eating) of PRS_BMI_ on obesity measures	0.00 (−0.01, 0.00)	0.2	0.00 (−0.01, 0.00)	0.2
Percentage mediation	—		—	
Mediation model through emotional and external eating				
Total effect of PRS_BMI_	0.29 (0.20, 0.38)	<0.001	0.24 (0.15, 0.32)	<0.001
Direct effect of PRS_BMI_	0.26 (0.19, 0.34)	<0.001	0.22 (0.15, 0.29)	<0.001
Indirect effect (via emotional and external eating) of PRS_BMI_ on obesity measures	0.03 (0.00, 0.05)	0.03	0.02 (0.00, 0.04)	0.1
Percentage mediation	10.3		—	

^1^Standardized regression coefficients (*β*) and 95% CIs. All models were adjusted for age, sex, and genetic principal components, and clustering was taken into account in all analyses. PRS_BMI_, polygenic risk score for BMI; WC, waist circumference.

**FIGURE 4:**
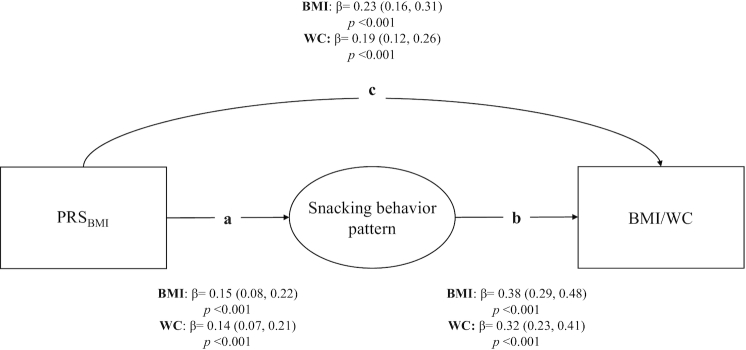
Results from the mediation model of the association between PRS_BMI_ and obesity measures. Standardized regression coefficients (95% CIs) from the mediation model of the snacking behavior pattern. All models were adjusted for age, sex, and genetic principal components, and twin pair clustering was taken into account in all analyses. The indirect effect (or mediation effect) is represented by ab, and c represents the direct effect; total effect = c + ab. Ellipses represent latent factors and rectangles represent observed variables. *n* = 949 for BMI and *n* = 874 for WC. PRS_BMI_, polygenic risk score for BMI; WC, waist circumference.

A more detailed description of the pathways of the mediation model for the snacking behavior pattern is shown in **Figure 4**. The PRS_BMI_ was positively associated with the snacking behavior pattern, indicating that individuals with a higher PRS_BMI_ are more susceptible to snacking (a: *β* = 0.15 for BMI and *β* = 0.14 for WC), and a higher loading on the snacking behavior pattern was associated with a higher BMI and WC (b: *β* = 0.38 and 0.32, respectively). These 2 pathways together represent the indirect effect, which was *β* = 0.06 for BMI and *β* = 0.05 for WC (Table 4).

When the analysis was limited to the mediation model through the DQS, there was no mediation in the association between the PRS_BMI_ and obesity measures (data not shown).

When analyzing men and women separately (Supplemental Table 3), we observed that the mediation model through the snacking behavior pattern was similar in men and women, although results were not statistically significant in men when we analyzed the mediation model of the association between the PRS_BMI_ and BMI. The infrequent and unhealthy eating behavior pattern significantly mediated the association between the PRS_BMI_ and BMI and WC in men but not in women, with higher mediation percentages (6.1% for BMI and 10.0% for WC) in men compared with the percentages in the overall sample (3.4% for BMI and 4.2% for WC). The mediation through the emotional and external eating behavior pattern was statistically significant in women for BMI. Despite these differences, when analyzing the models through the snacking, the infrequent and unhealthy, and the emotional and external eating behavior patterns, the 95% CIs were overlapping in both men and women.

The PRS_BMI_ was not associated with WC adjusted for BMI (data not shown). The associations between the PRS_WHR_ and WC adjusted for BMI are shown in **Supplemental Table 4**. Despite an overall association between the 4 eating behavior patterns and WC adjusted for BMI, eating behavior patterns did not mediate any of these pathways.

## Discussion

In this cross-sectional study of healthy young adults, we showed that the association between the most recent PRS_BMI_ and obesity measures was partly mediated by the snacking behavior pattern in men and women and to a lesser extent by the infrequent and unhealthy eating behavior pattern in men. The association between the PRS_BMI_ and BMI was also partly mediated by the emotional and external eating pattern. In the twin modeling, the associations between the snacking and the emotional and external eating behavior patterns and BMI and WC were largely explained by genetic factors, supporting the inference that the snacking and the emotional and external eating behavior patterns and obesity share a common underlying genetic architecture. Another important finding was that eating behavior patterns were moderately heritable, with the remaining variance accounted for by nonshared environmental factors.

Our results are novel because, to the best of our knowledge, research to date has not yet employed polygenetic risk and classical twin modeling simultaneously to study the genetic architecture underlying eating behavior patterns and obesity. Because the mediation by snacking has not been reported previously, it is difficult to compare our results with the previous literature. A recent review from Vainik et al. (46) supported that uncontrolled eating might involve other eating behavior constructs, which include the overeating behavior. Our snacking behavior pattern had a high loading on alternating restriction and overeating style, somewhat similar to these previous constructs (46). Our results are largely consistent with other studies using a PRS_BMI_ based on <100 SNPs, that have previously reported mediation by undesirable eating behavior traits (21–23). In line with our findings, 2 previous studies have shown that the association between a 90-loci PRS for obesity and obesity measures was partially mediated by uncontrolled eating and emotional eating (21, 22). A more recent study reported similar results for disinhibition and susceptibility to hunger, a behavior similar to uncontrolled eating, as a mediator of the genetic susceptibility to obesity (23). Emotional eating was identified as a mediator of the association between a 90-loci PRS for obesity and BMI and WC in the study by Konttinen et al. (21). In that study, the indirect association had a *β* coefficient of 0.02, and the total effect was *β* = 0.14 and 0.17 for BMI and WC, respectively, which are similar in the present study, although our results were not significant for WC. Previous studies, including that by Konttinen et al., mainly assessed eating behaviors with different versions of the Three-Factor Eating Questionnaire (28, 29), whereas we used a questionnaire that was specifically developed for the FT16 study (27, 32). Another feature specific to our study is that, in addition to eating behaviors, we also included information about diet quality, which was assessed by a previously validated questionnaire (32). Furthermore, all of the previous studies used a PRS_BMI_ with <100 loci to capture genetic susceptibility to obesity, whereas we used the latest PRS by incorporating all available information regardless of the significance level (24).

Both WHR and WC adjusted for BMI have been suggested as indirect measures of abdominal obesity (47). Previous research has shown that a PRS_WHR_ is not associated with obesity measures (48), most likely because genetic variants associated with WHR are highly expressed in the adipose tissue (49) instead of the brain (19), making mediation by appetitive traits less plausible. In line, in the present study, the PRS_BMI_ was not associated with WC adjusted for BMI. Further, eating behavior patterns did not mediate the associations between the PRS_WHR_ and any of the obesity measures.

In the present study, we found that most of the eating behavior patterns were significantly associated with obesity measures, similarly to previous studies (13, 50, 51). The strongest associations were seen with the snacking behavior pattern and the emotional and external eating behavior pattern, and genetic factors were largely underlying these associations. These findings accord with other studies on other eating behavior traits, such as that by Keskitalo et al. (13), who showed that the associations between uncontrolled eating and BMI as well as emotional eating and BMI are mostly explained by genetic factors. Similarly, twin studies investigating the intake of unhealthy foods and beverages, such as fast food (52) and soda (53), have shown associations with BMI—which are largely explained by genetic factors common to both. Therefore, together with previous research, our results suggest that eating behavior patterns share a common genetic liability with obesity measures.

Eating behavior patterns were moderately heritable, suggesting that they are influenced by both genetic and environmental factors. In general, our heritability estimates of eating behavior patterns appeared to be somewhat lower than those measured with the Three-Factor Eating Questionnaire (13, 51). In the present study, we found that the emotional and external eating behavior pattern was moderately heritable, similar to the female Finnish sample from Keskitalo et al. (13) (*h*^2^ = 31%). Another study showed heritability estimates of ∼43% for restrained eating (50), somewhat similar to avoidant eating in our study, which included avoiding greasy meals and avoiding calories eating style. Our heritability estimates for BMI are consistent with a large-scale meta-analysis and pooled analyses on BMI (10–12).

Previous research suggests that genetic factors from heritability estimates provide more evidence for the genetic contribution to obesity susceptibility than genetic variants from GWASs, due to the large difference in the percentage of variance explained between them (54). Twin studies have demonstrated that a large proportion of the variance in BMI as well as appetitive traits is explained by genetic factors in childhood and adulthood (2, 10, 11, 55). GWASs have recently increased the knowledge base of common genetic variants associated with obesity (17), and the PRS used in this study explained 3 times as much of the variance (8.3%) than the PRS reported in previous studies (3%) (21–23). In summary, based on the most recent and comprehensive PRS to date, our results strongly suggest that obesity susceptibility genes influence eating behavior patterns and obesity traits consequently.

Our study has several potential limitations. The main limitation is its cross-sectional design; we therefore cannot confirm the causal directions of the relations between eating behavior patterns and obesity measures. However, a previous longitudinal study tested the reciprocity between restrained eating and BMI and WC to determine their potential relations, and they indicated that restrained eating was a marker for previous weight gain instead of a trigger of future weight gain (56). Future research is needed to carefully test the direction of these relations in a longitudinal design. Despite this, the key question of this study was to examine the genetic architecture underlying obesity measures and eating behavior patterns. We focused on the role of genes rather than the causal associations between obesity measures and eating behavior patterns. Furthermore, the eating styles questionnaire used in this study is a short tool, specifically developed for the FT16 cohort, and therefore is less detailed than other well-established long questionnaires. In this study, we were interested in the overall eating style of the study participants rather than the cognitive aspects of eating and eating behaviors that are assessed by the Three-Factor Eating Questionnaire (28, 29), used in previous similar studies (21–23). Moreover, previous studies that have used this questionnaire in the same cohort have shown associations between these eating styles with obesity (27) as well as diet quality (32). As in all studies with self-reported data, a further limitation of this study is that eating styles, diet quality, and obesity measures were self-reported by the participants, therefore misreporting might have occurred, especially in women and obese people, because they tend to underreport nutrient intakes and behaviors considered unhealthy (57, 58). Finally, the generalizability of these results is subject to certain limitations because we used a Finnish cohort of young adults with European ancestry. Further studies are needed to test the generalizability of these results in other non-Westernized populations.

Our study has some important strengths. To our knowledge, this is the first study in adults that tested whether the genetic susceptibility to obesity is mediated by eating behaviors by using a PRS that included almost 1 million SNPs representing the whole genome. We included a combination of twin modeling with GWASs analyses based on very different underlying assumptions. The robustness of our results based on these different methods provides further evidence on the common genetic background of eating behaviors and obesity measures. Finally, the inclusion of diet quality in addition to eating behavior patterns allowed us to assess an additional aspect of diet.

In conclusion, our findings support that eating behavior patterns are moderately heritable. Snacking, and to a lesser extent infrequent and unhealthy and emotional and external eating behavior patterns, share a common genetic liability with obesity measures. Obesity prevention efforts might benefit from focusing on eating behavior change, particularly in genetically susceptible individuals. Future longitudinal and intervention studies will be beneficial to understand how eating behavior patterns and genetic susceptibility to obesity impact life-long weight outcomes.

## Supplementary Material

nqaa181_Supplemental_File

## Notes

GM has received funding from the University of Helsinki Faculty of Medicine PhD program, and the Juho Vainio Foundation. AK-R has been supported by the Gyllenberg Foundation. PNS has been supported by the Helsinki Institute of Life Science and NordFosk during the conduct of the study, and by the Finnish Foundation for Alcohol Studies, outside the submitted work. LHB acknowledges funding from the Austrian Science Fund (FWF): M 2449. JK has been supported by the Academy of Finland (grant #312073). Formation, data collection, and maintenance of the FT16 cohort has been supported by the National Institute of Alcohol Abuse and Alcoholism (grants AA-12502 and AA-00145 to R J Rose, Indiana University) and the Academy of Finland (grants 100499, 205585, and 118555 to JK).

Data described in the manuscript, code book, and analytic code will be made available upon request pending application, approval with a material transfer agreement between the University of Helsinki and the applicant's host institution in accordance with current data protection legislation.

Supplemental Methods, Supplemental Tables 1–4, and Supplemental Figures 1 and 2 are available from the “Supplementary data” link in the online posting of the article and from the same link in the online table of contents at https://academic.oup.com/ajcn/.

Abbreviations used: A, additive genetic variation; C, shared environmental variation; D, dominance genetic variation; DQS, diet quality score; DZ, dizygotic; E, nonshared environmental variation; FT16, FinnTwin16; GWAS, genome-wide association study; ICC, intraclass correlation; LD, linkage disequilibrium; MAF, minor allele frequency; MZ, monozygotic; OS-DZ, opposite-sex dizygotic; PCA, principal component analysis; PRS, polygenic risk score; SEM, structural equation modeling; SNP, single nucleotide polymorphism; SS-DZ, same-sex dizygotic; WC, waist circumference; WHR, waist-to-hip ratio.
