# Supplementary material for: The genetic architecture of the association between eating behaviors and obesity: combining genetic twin modeling and polygenic risk scores
Source: Am J Clin Nutr. 2020 Jul 20;112(4):956–66. doi: 10.1093/ajcn/nqaa181 (PMC7528566; doi:10.1093/ajcn/nqaa181)
Supplement: nqaa181_Supplemental_File [file nqaa181_supplemental_file.docx]

**On-line supporting material**

**The genetic architecture of the association between eating behaviors and obesity: combining genetic twin modeling and polygenic risk scores**

Guiomar Masip^1^, Karri Silventoinen^1,2^, Anna Keski-Rahkonen^1^, Teemu Palviainen^3^, Pyry N. Sipilä^1^, Jaakko Kaprio^1,3^, Leonie H. Bogl^3,4*^

^1^Department of Public Health, University of Helsinki, Helsinki, Finland

^2^Population Research Unit, Faculty of Social Sciences, University of Helsinki, Helsinki, Finland

^3^Institute for Molecular Medicine Finland (FIMM), University of Helsinki, Helsinki, Finland

^4^Department of Epidemiology, Center for Public Health, Medical University of Vienna, Vienna, Austria

***Supplementary methods***

*Genotyping and Quality Control*

Genotyping was done using Illumina Human610-Quad v1.0 B and Human670-QuadCustom v1.0 arrays at the Wellcome Trust Sanger Institute (Cambridge UK), Illumina HumanCoreExome (12 v1.0 A, 12 v1.1 A, 24 v1.0 A, 24 v1.1 A, 24 v1.2 A) arrays at the Broad Institute of MIT and Harvard (Boston, MA, USA), Wellcome Trust Sanger Institute (Cambridge, UK), University of Chicago Genomics Facility (Chicago IL, USA) and Institute for Molecular Medicine Finland (Helsinki, Finland) and the Affymetrix FinnGen Axiom array at Thermo Fisher Scientific (Santa Clara, CA, USA). The algorithms for genotype calling were Illumina’s GenCall for all HumanCoreExome chip genotypes, Illuminus for 610k & 670k chip genotypes and AxiomGT1 for Affymetrix chip genotypes. On Illumina arrays where genotypes were called to Illumina’s TOP strand, strands were flipped to forward strand using strands files generated by Will Rayner (see URLs).

The genome build of all genotypes were set to GRCh37/hg19. In cases where genotypes were called to NCBI36/hg18 or GRCh38/hg38, genome positions were lifted to GRCh37/hg19 using the University of California Santa Cruz LiftOver program (1) with appropriate chain file. Genotype quality control was done in three batches (batch 1: 610k+670k, batch 2: HumanCoreExome and batch 3: Affymetrix chip genotypes). First, we removed every genetic variant with a call rate <0.975 (batches 1 and 3) or <0.95 (batch 2). Second, we removed every sample with a call rate <0.98 (batch 1) or <0.95 (batches 2 and 3). Finally, variants were filtered by their minor allele frequency (MAF) <0.01 with Hardy-Weinberg Equilibrium p-value <1e-06. Samples from all batches with heterozygosity test method-of-moments F coefficient estimate values <-0.03 or >0.05 (batches 1 and 2) or ±4SD from the mean (batch 3) were removed, along with the samples which failed the sex check or were among the multi-dimensional scaling principal component analysis outliers. The total amount of genotyped autosomal variants after quality control were 475,526 (batch 1), 239,894 (batch 2) and 388,673 (batch 3), with the following number of samples remaining for imputation: 2,617 (batch 1), 5,328 (batch 2) and 8,218 (batch 3). We performed pre-phasing using Eagle v2.3 (2) and imputation with Minimac3 v2.0.1 using the University of Michigan Imputation Server (3). Genotypes from all batches were imputed to the Haplotype Reference Consortium release 1.1 reference panel (4). The final study sample was extracted from the data, where all batches of imputed data were merged.

*Polygenic scoring*

Genetic susceptibility was assessed by calculating PRSes using the Bayesian approach accounting for linkage disequilibrium (LD) between each genetic variant, which is used to infer the posterior mean effect sizes along with the LD information from an external reference panel (5). Therefore, we did not use any pruning and thresholding method to select genetic variants. The infinitesimal model for polygenic scoring was adjusted by an LD reference panel consisting of the Finnish FINRISK study samples (n = 27,284) (6). GWAS meta-analysis summary statistics for BMI were obtained from the GIANT consortium website (7). GWAS summary statistics for waist-to-hip ratio (WHR) adjusted for BMI were obtained from the data repository provided by Pulit et al. (8). GWAS summary statistics, the LD reference panel and the FT16 study sample were restricted to the European HapMap3 variants (9) with an MAF >5%. The major histocompatibility complex (MHC) gene cluster of human chromosome 6 (GRCh37: 6p22.1-21.3) was excluded due to a strong LD block. We derived two PRSes, one for BMI and one for WHR adjusted for BMI. The total number of genetic variants used for the PRS calculations were 996,919 for BMI and 1,148,565 for WHR adjusted for BMI, with the re-weighted effect sizes available from 692,578 and 484,563 samples, respectively. The PRS_BMI_ explained 8.9% of the BMI variance in this study sample.

**REFERENCES**

1. Hinrichs AS, Karolchik D, Baertsch R, Barber GP, Bejerano G, Clawson H, Diekhans M, Furey TS, Harte RA. The UCSC Genome Browser Database: update 2006. Nucleic Acids Res. 2006;34:D590–8.

2. Loh PR, Danecek P, Palamara PF, Fuchsberger C, Reshef YA, Finucane HK, Schoenherr S, Forer L, McCarthy S, Abecasis GR, et al. Reference-based phasing using the Haplotype Reference Consortium panel. Nat Genet. 2016;48:1443–8.

3. Das S, Forer L, Schönherr S, Sidore C, Locke AE, Kwong A, Vrieze SI, Chew EY, Levy S, McGue M, et al. Next-generation genotype imputation service and methods. Nat Genet. 2016;48:1284–7.

4. McCarthy S, Das S, Kretzschmar W, Delaneau O, Wood AR, Teumer A, Kang HM, Fuchsberger C, Danecek P, Sharp K, et al. A reference panel of 64,976 haplotypes for genotype imputation. Nat Genet. 2016;48:1279–83.

5. Vilhjálmsson BJ, Yang J, Finucane HK, Gusev A, Lindström S, Ripke S, Genovese G, Loh PR, Bhatia G, Do R, et al. Modeling Linkage Disequilibrium Increases Accuracy of Polygenic Risk Scores. Am J Hum Genet. 2015;97:576–92.

6. Borodulin K, Vartiainen E, Peltonen M, Jousilahti P, Juolevi A, Laatikainen T, Männistö S, Salomaa V, Sundvall J, Puska P. Forty-year trends in cardiovascular risk factors in Finland. Eur J Public Health. 2015;25:539–46.

7. Yengo L, Sidorenko J, Kemper KE, Zheng Z, Wood AR, Weedon MN, Frayling TM, Hirschhorn J, Yang J, Visscher PM. Meta-analysis of genome-wide association studies for height and body mass index in ∼ 700 000 individuals of European ancestry. Hum Mol Genet. 2018;27:3641–9.

8. Pulit SL, Stoneman C, Morris AP, Wood AR, Glastonbury CA, Tyrrell J, Yengo L, Ferreira T, Marouli E, Ji Y, et al. Meta-Analysis of genome-wide association studies for body fat distribution in 694 649 individuals of European ancestry. Hum Mol Genet. 2019;28:166–74.

9. Altshuler DM, Gibbs RA, Peltonen L, Schaffner SF, Yu F, Dermitzakis E, Bonnen PE, De Bakker PIW, Deloukas P, Gabriel SB, et al. Integrating common and rare genetic variation in diverse human populations. Nature. 2010;467:52–8.

**URLs**

Will Rayner's strand files. Internet: <https://www.well.ox.ac.uk/~wrayner/strand/>

**Supplemental Table 1A.** General characteristics of the study sample in twin individuals by zygosity

| **Characteristics** | **MZ twins**  **(n = 1,298)** | | **SS-DZ twins**  **(n = 1,272)** | | **OS-DZ twins**  **(n = 1,407)** | |
| --- | --- | --- | --- | --- | --- | --- |
|  | Men  (n = 505) | Women  (n = 793) | Men  (n = 603) | Women  (n = 669) | Men  (n = 624) | Women  (n = 783) |
| Age, y, mean (95% CI) | 34.1 (33.9, 34.2) | 34.1 (33.9, 34.2) | 34.1 (34.0, 34.2) | 34.1 (34.0, 34.2) | 34.2 (34.1, 34.3) | 34.1 (34.1, 34.2) |
| BMI in kg/m^2^, mean (95% CI) | 25.6 (25.3, 25.9) | 23.8 (23.4, 24.2) | 25.8 (25.5, 26.1) | 24.3 (23.9, 24.7) | 25.8 (25.5, 26.1) | 23.9 (23.6, 24.2) |
| Obesity (BMI >30kg/m^2^), n (%) | 50 (9.9) | 78 (9.8) | 71 (11.1) | 81 (12.1) | 65 (10.4) | 70 (8.9) |
| WC in cm, mean (95% CI)^1^ | 91.6 (90.4, 92.9) | 80.5 (79.5, 81.6) | 92.4 (91.5, 93.3) | 82.1 (81.1, 83.1) | 92.3 (91.5, 93.1) | 81.4 (80.5, 82.2) |
| Abdominal obesity (Women >88 cm; Men >102 cm), n (%)^1^ | 73 (14.9) | 154 (20.6) | 98 (16.4) | 165 (26.3) | 100 (15.9) | 190 (24.6) |
| Diet quality score, mean (95% CI) | 6.4 (6.2, 6.6) | 7.3 (7.2, 7.5) | 6.4 (6.2, 6.5) | 7.4 (7.2, 7.6) | 6.5 (6.3, 6.6) | 7.5 (7.3, 7.6) |
| Standardized PRS_BMI_, mean (95% CI)^1^ | 0.0 (-0.1, 0.2) | 0.0 (-0.2, 0.1) | 0.0 (-0.1, 0.2) | 0.0 (-0.2, 0.1) | 0.0 (-0.3, 0.2) | 0.0 (-0.2, 0.2) |
| Standardized PRS_WHR_, mean (95% CI)^1^ | 0.0 (-0.1, 0.2) | 0.0 (-0.2, 0.1) | 0.0 (-0.1, 0.2) | 0.0 (-0.2, 0.1) | 0.0 (-0.2, 0.3) | 0.0 (-0.3, 0.2) |

Abbreviations: body mass index (BMI); monozygotic (MZ); opposite-sex dizygotic (OS-DZ); polygenic risk score (PRS); same-sex dizygotic (SS-DZ); waist circumference (WC); waist-to-hip ratio (WHR). ^1^Sample size smaller due to missing values.

**Supplemental Table 1B.** General characteristics of the study sample for mediation analyses

| **Characteristics** | **Overall**  **(n = 949)** | **Men**  **(n = 410)** | **Women**  **(n = 539)** | *P-value* |
| --- | --- | --- | --- | --- |
| Monozygotic twins, n (%) | 445 (46.9) | 198 (48.3) | 247 (45.8) | 0.6 |
| Same-sex dizygotic twins, n (%) | 353 (37.2) | 144 (35.1) | 209 (38.8) |  |
| Opposite-sex dizygotic twins, n (%) | 151 (15.9) | 68 (16.6) | 83 (15.4) |  |
| Age, y, mean (95% CI) | 34.0 (33.9, 34.1) | 34.0 (33.8, 34.1) | 34.0 (33.9, 34.2) | 0.5 |
| BMI in kg/m^2^, mean (95% CI) | 24.9 (24.6, 25.2) | 25.7 (25.3, 26.2) | 24.2 (23.8, 24.7) | <0.001 |
| Obesity (BMI >30kg/m^2^), n (%) | 111 (11.7) | 48 (11.7) | 63 (11.7) | <1.0 |
| WC in cm, mean (95% CI)^1^ | 86.4 (85.3, 87.4) | 91.4 (90.1, 92.8) | 82.1 (80.7, 83.5) | <0.001 |
| Abdominal obesity (Women >88 cm; Men >102 cm), n (%)^1^ | 184 (21.2) | 65 (16.3) | 119 (25.1) | 0.005 |
| Diet quality score, mean (95% CI) | 7.1 (6.9, 7.2) | 6.5 (6.3, 6.7) | 7.5 (7.3, 7.7) | <0.001 |

Abbreviations: body mass index (BMI); waist circumference (WC). Differences between men and women, *p*-values, were determined by the adjusted Wald test for continuous variables and Pearson’s χ^2^ test for categorical variables and corrected for the clustering of twin pairs by survey methods. ^1^Sample size smaller due to missing values. Overall sample size for WC and abdominal obesity n = 874.

**Supplemental Table 2.** Intraclass correlations for eating behavior patterns and obesity measures

|  | MZ correlation coefficient (95% CI)  (n = 527 pairs) | SS-DZ correlation coefficient (95% CI) (n = 470 pairs) | OS-DZ correlation coefficient (95% CI) (n = 503 pairs) |
| --- | --- | --- | --- |
| **Snacking** | 0.41 (0.34, 0.48) | 0.14 (0.05, 0.22) | 0.10 (0.01, 0.19) |
| **Infrequent and unhealthy eating** | 0.51 (0.45, 0.58) | 0.15 (0.06, 0.23) | 0.19 (0.10, 0.27) |
| **Avoidant eating** | 0.36 (0.28, 0.43) | 0.15 (0.06, 0.24) | 0.13 (0.04, 0.21) |
| **Emotional and external eating** | 0.36 (0.29, 0.44) | 0.24 (0.15, 0.32) | 0.01 (0.00, 0.10) |
| **BMI** | 0.76 (0.72, 0.80) | 0.37 (0.30, 0.45) | 0.20 (0.12, 0.29) |
| **WC**^1^ | 0.64 (0.59, 0.69) | 0.28 (0.20, 0.37) | 0.17 (0.08, 0.25) |

Intraclass correlations and 95% CIs. All models were adjusted for age and sex. Abbreviations: body mass index (BMI); monozygotic (MZ); opposite-sex dizygotic (OS-DZ); same-sex dizygotic (SS-DZ); waist circumference (WC). ^1^Sample size smaller due to missing values.

**Supplemental Figure 1.** Proportion of variation of the eating behavior patterns and obesity measures explained by additive genetics (A) and non-shared environmental factors (E) for men and women.

Proportion of variance explained

0.34

(0.22,

0.45)

0.42

(0.33,

0.49)

0.66

(0.55,

0.78)

0.58

(0.51,

0.67)

0.45

(0.35,

0.54)

0.50

(0.42,

0.57)

0.55

(0.46,

0.65)

0.50

(0.43,

0.58)

0.33

(0.21,

0.44)

0.37

(0.28,

0.45)

0.67

(0.56,

0.79)

0.63

(0.55,

0.72)

0.33

(0.22,

0.43)

0.38

(0.30,

0.45)

0.67

(0.57,

0.78)

0.62

(0.55,

0.70)

0.76

(0.70,

0.80)

0.77

(0.73,

0.81)

0.24

(0.20,

0.30)

0.23

(0.19,

0.27)

0.60

(0.51,

0.69)

0.64

(0.58,

0.69)

0.40

(0.33,

0.49)

0.36

(0.31,

0.42)

The numbers within the bars are means and their 95% CIs. Abbreviations: body mass index (BMI); waist circumference (WC). n = 1,325 for men and n = 1,675 for women.

**Supplemental Figure 2.** Results from the mediation model of the association between PRS_BMI_ and obesity measures

PRS_BMI_

Infrequent and unhealthy eating behavior pattern

BMI/WC

**BMI**: *β* = 0.28 (0.20, 0.36)

*p* <0.001

**WC:** *β* = 0.22 (0.15, 0.30)

*p* <0.001

**BMI**: *β* = 0.09 (0.02, 0.16)

*p* = 0.008

**WC**: *β* = 0.09 (0.02, 0.16)

*p* = 0.01

**BMI**: *β* = 0.12 (0.05, 0.20)

*p* = 0.001

**WC:** *β* = 0.14 (0.07, 0.20)

*p* <0.001

**c**

**b**

**a**

# Standardized regression coefficients (95% CIs) from the mediation model of infrequent and unhealthy eating behavior pattern. All models were adjusted for age, sex and genetic principal components, and twin pair clustering was taken into account in all analyses. ab represents the indirect effect (or mediation effect) and c represents the direct effect, total effect = c + ab. Ellipses represent latent factors and rectangles represent observed variables. Abbreviations: body mass index (BMI), polygenic risk score for BMI (PRS_BMI_), waist circumference (WC). n = 949 for BMI and n = 874 for WC.

**Supplemental Table 3.** Structural equation modeling of the PRS_BMI_ and obesity measures for men and women

|  | BMI for men(n = 410) |  | BMI for women (n = 539) |  | WC for men(n = 399) |  | WC for women (n = 475) |  |
| --- | --- | --- | --- | --- | --- | --- | --- | --- |
|  | *β* (95% CIs) | *P-value* | *β* (95% CIs) | *P-value* | *β* (95% CIs) | *P-value* | *β* (95% CIs) | *P-value* |
| Mediation model through snacking |  |  |  |  |  |  |  |  |
| Total effect of PRS_BMI_^1^ | 0.33 (0.18, 0.47) | <0.001 | 0.24 (0.15, 0.33) | <0.001 | 0.30 (0.16, 0.43) | <0.001 | 0.18 (0.09, 0.28) | <0.001 |
| Direct effect of PRS_BMI_^2^ | 0.27 (0.17, 0.37) | <0.001 | 0.19 (0.11, 0.28) | <0.001 | 0.24 (0.14, 0.34) | <0.001 | 0.15 (0.06, 0.24) | 0.002 |
| Indirect effect (via snacking eating) of PRS_BMI_ on obesity measures^3^ | 0.06 (0.00, 0.11) | 0.05 | 0.05 (0.01, 0.09) | 0.007 | 0.06 (0.00, 0.11) | <0.05 | 0.04 (0.00, 0.07) | 0.03 |
| % mediation | - |  | 20.8 |  | 20.0 |  | 22.2 |  |
| Mediation model through infrequent and unhealthy eating |  |  |  |  |  |  |  |  |
| Total effect of PRS_BMI_^1^ | 0.33 (0.18, 0.47) | <0.001 | 0.24 (0.15, 0.33) | <0.001 | 0.30 (0.16, 0.43) | <0.001 | 0.18 (0.09, 0.28) | <0.001 |
| Direct effect of PRS_BMI_^2^ | 0.31 (0.17, 0.44) | <0.001 | 0.24 (0.15, 0.33) | <0.001 | 0.27 (0.14, 0.40) | <0.001 | 0.18 (0.09, 0.27) | <0.001 |
| Indirect effect (via infrequent and unhealthy eating) of PRS_BMI_ on obesity measures^3^ | 0.02 (0.00, 0.04) | 0.04 | 0.00 (-0.01, 0.02) | 0.4 | 0.03 (0.01, 0.05) | 0.02 | 0.00 (-0.01, 0.02) | 0.5 |
| % mediation | 6.1 |  | - |  | 10.0 |  | - |  |
| Mediation model through avoidant eating |  |  |  |  |  |  |  |  |
| Total effect of PRS_BMI_^1^ | 0.33 (0.18, 0.47) | <0.001 | 0.24 (0.15, 0.33) | <0.001 | 0.30 (0.16, 0.43) | <0.001 | 0.18 (0.09, 0.28) | <0.001 |
| Direct effect of PRS_BMI_^2^ | 0.32 (0.18, 0.47) | <0.001 | 0.26 (0.17, 0.35) | <0.001 | 0.30 (0.16, 0.43) | <0.001 | 0.20 (0.10, 0.30) | <0.001 |
| Indirect effect (via avoidant eating) of PRS_BMI_ on obesity measures^3^ | 0.00 (-0.01, 0.01) | 0.8 | -0.01 (-0.03, 0.00) | 0.09 | 0.00 (0.00, 0.01) | <0.9 | -0.01 (-0.03, 0.00) | 0.13 |
| % mediation | - |  | - |  | - |  | - |  |
| Mediation model through emotional and external eating |  |  |  |  |  |  |  |  |
| Total effect of PRS_BMI_^1^ | 0.33 (0.18, 0.47) | <0.001 | 0.24 (0.15, 0.33) | <0.001 | 0.30 (0.16, 0.43) | <0.001 | 0.18 (0.09, 0.28) | <0.001 |
| Direct effect of PRS_BMI_^2^ | 0.31 (0.19, 0.43) | <0.001 | 0.21 (0.13, 0.29) | <0.001 | 0.28 (0.17, 0.40) | <0.001 | 0.16 (0.07, 0.25) | <0.001 |
| Indirect effect (via emotional and external eating) of PRS_BMI_ on obesity measures^3^ | 0.02 (-0.02, 0.05) | 0.4 | 0.04 (0.00, 0.07) | 0.03 | 0.02 (-0.02, 0.05) | 0.4 | 0.02 (-0.01, 0.05) | 0.12 |
| % mediation | - |  | 16.7 |  | - |  | - |  |

# Standardized regression coefficients (*β*) and 95% confidence intervals (CIs). All models were adjusted for age and genetic principal components, and clustering was taken into account in all analyses. ^1^Total effect = c + ab; ^2^direct effect = c; ^3^indirect effect = ab. Abbreviations: body mass index (BMI); polygenic risk score for BMI (PRS_BMI_); waist circumference (WC).

**Supplemental Table 4.** Structural equation modeling of the PRS_WHR_ and obesity measures

|  | BMI(n = 949) |  | WC(n = 874) |  | **WC adjusted for BMI (n = 874)** |  |
| --- | --- | --- | --- | --- | --- | --- |
|  | *β* (95% CIs) | *P-value* | *β* (95% CIs) | *P-value* | *β* (95% CIs) | *P-value* |
| Mediation model through snacking |  |  |  |  |  |  |
| Total effect of PRS_WHR_^1^ | 0.02 (-0.07, 0.11) | 0.6 | 0.07 (-0.02, 0.15) | 0.1 | 0.10 (0.03, 0.16) | 0.007 |
| Direct effect of PRS_WHR_^2^ | -0.01 (-0.08, 0.07) | 0.9 | 0.04 (-0.04, 0.11) | 0.3 | 0.09 (0.02, 0.16) | 0.008 |
| Indirect effect (via snacking eating) of PRS_WHR_ on obesity measures^3^ | 0.03 (0.00, 0.06) | 0.08 | 0.03 (0.00, 0.06) | 0.06 | 0.00 (0.00, 0.01) | 0.6 |
| Mediation model through infrequent and unhealthy eating |  |  |  |  |  |  |
| Total effect of PRS_WHR_^1^ | 0.02 (-0.07, 0.11) | 0.6 | 0.07 (-0.02, 0.15) | 0.1 | 0.10 (0.03, 0.16) | 0.007 |
| Direct effect of PRS_WHR_^2^ | 0.01 (-0.08, 0.10) | 0.8 | 0.05 (-0.03, 0.13) | 0.2 | 0.09 (0.02, 0.16) | 0.01 |
| Indirect effect (via infrequent and unhealthy eating) of PRS_WHR_ on obesity measures^3^ | 0.01 (0.00, 0.03) | 0.09 | 0.01 (0.00, 0.03) | 0.06 | 0.00 (0.00, 0.01) | 0.3 |
| Mediation model through avoidant eating |  |  |  |  |  |  |
| Total effect of PRS_WHR_^1^ | 0.02 (-0.07, 0.11) | 0.6 | 0.07 (-0.02, 0.15) | 0.1 | 0.10 (0.03, 0.16) | 0.007 |
| Direct effect of PRS_WHR_^2^ | 0.02 (-0.07, 0.11) | 0.6 | 0.07 (-0.02, 0.15) | 0.1 | 0.10 (0.03, 0.16) | 0.007 |
| Indirect effect (via avoidant eating) of PRS_WHR_ on obesity measures^3^ | 0.00 (0.00, 0.00) | 0.8 | 0.00 (0.00, 0.01) | 0.7 | 0.00 (0.00, 0.00) | 0.8 |
| Mediation model through emotional and external eating |  |  |  |  |  |  |
| Total effect of PRS_WHR_^1^ | 0.02 (-0.07, 0.11) | 0.6 | 0.07 (-0.02, 0.15) | 0.1 | 0.10 (0.03, 0.16) | 0.007 |
| Direct effect of PRS_WHR_^2^ | 0.01 (-0.08, 0.09) | 0.9 | 0.05 (-0.03, 0.13) | 0.2 | 0.10 (0.03, 0.17) | 0.007 |
| Indirect effect (via emotional and external eating) of PRS_WHR_ on obesity measures^3^ | 0.02 (-0.01, 0.04) | 0.2 | 0.01 (-0.01, 0.04) | 0.2 | 0.00 (-0.01, 0.00) | 0.7 |

# Standardized regression coefficients (*β*) and 95% confidence intervals (CIs). All models were adjusted for age and sex, and clustering was taken into account in all analyses. ^1^Total effect = c + ab; ^2^direct effect = c; ^3^indirect effect = ab. Abbreviations: body mass index (BMI); polygenic risk score for WHR adjusted for BMI (PRS_WHR_), waist circumference (WC), waist-to-hip ratio (WHR).
